# Neurotensin and CRH Interactions Augment Human Mast Cell Activation

**DOI:** 10.1371/journal.pone.0048934

**Published:** 2012-11-14

**Authors:** Konstantinos–Dionysios Alysandratos, Shahrzad Asadi, Asimenia Angelidou, Bodi Zhang, Nikolaos Sismanopoulos, Hailing Yang, Agatha Critchfield, Theoharis C. Theoharides

**Affiliations:** 1 Molecular Immunopharmacology and Drug Discovery Laboratory, Department of Molecular Physiology and Pharmacology, Tufts University School of Medicine, Boston, Massachusetts, United States of America; 2 Allergy Clinical Research Center, Allergy Section, Attikon General Hospital, University of Athens Medical School, Athens, Greece; 3 Department of Pharmacy, Tufts Medical Center, Boston, Massachusetts, United States of America; 4 Sackler School of Graduate Biomedical Sciences, Tufts University, Boston, Massachusetts, United States of America; 5 Division of Maternal/Fetal Medicine, Department of Obstetrics and Gynecology, Tufts University School of Medicine and Tufts Medical Center, Boston, Massachusetts, United States of America; 6 Department of Biochemistry, Tufts University School, Boston, Massachusetts, United States of America; 7 Department of Internal Medicine, Tufts University School of Medicine and Tufts Medical Center, Boston, Massachusetts, United States of America; 8 Department of Psychiatry, Tufts University School of Medicine and Tufts Medical Center, Boston, Massachusetts, United States of America; Statens Serum Institute, Denmark

## Abstract

Stress affects immunity, but the mechanism is not known. Neurotensin (NT) and corticotropin-releasing hormone (CRH) are secreted under stress in various tissues, and have immunomodulatory actions. We had previously shown that NT augments the ability of CRH to increase mast cell-dependent skin vascular permeability in rodents. Here we show that NT triggered human mast cell degranulation and significantly augmented CRH-induced vascular endothelial growth factor (VEGF) release. Investigation of various signaling molecules indicated that only NF-κB activation was involved. These effects were blocked by pretreatment with the NTR antagonist SR48692. NT induced expression of CRH receptor-1 (CRHR-1), as shown by Western blot and FACS analysis. Interestingly, CRH also induced NTR gene and protein expression. These results indicate unique interactions among NT, CRH, and mast cells that may contribute to auto-immune and inflammatory diseases that worsen with stress.

## Introduction

Stress affects immunity, but its mechanism is not understood. Neurotensin (NT) is a vasoactive peptide originally isolated from the brain [1] and has been implicated in immunity [2], [3], but its role in the stress response has not been investigated. NT is increased in the skin following acute stress, stimulates skin mast cells and increases vascular permeability in rodents [4]. NT administration increases vascular permeability in isolated rat skin [5] and in skin blisters through mast cell activation [6]. NT also stimulates rodent mast cells to secrete histamine and elevates histamine plasma levels through NTR [7]–[9]. Moreover, NT is rapidly degraded by mast cell proteases [10], [11] implying tight regulation of its activity.

Activation of mast cells leads to release of multiple mediators with potent vasodilatory, inflammatory and nociceptive properties [12] through which they participate in acute and delayed hypersensitivity reactions in the skin [13]. In addition, mast cells are critical for innate and acquired immunity [14], as well as for inflammatory processes [12], [15]. In fact, skin mast cells may function as “sensors” of environmental and emotional stress, possibly through direct activation by corticotropin-releasing hormone (CRH) and related peptides [16].

There is considerable evidence that stress worsens allergic diseases in general [17]–[20], as well as skin diseases such as atopic dermatitis (AD) [21]–[24]. AD is characterized by chronic inflammation and severe pruritus [25]–[29], and involves increased mast cells in AD lesions [30], [31]. Moreover, there are increased nerve-mast cell contacts in the skin of AD patients [32], implying the possible effect of neuropeptides on mast cells. However, even though some neuropeptides have been studied in AD skin [33], there was no significant pattern and NT was not investigated. We had previously shown that acute restraint stress in rats stimulates degranulation of skin mast cells, and increases skin vascular permeability, an effect mimicked by intradermal injection of CRH [34]. We had also shown that NT augments the effect of stress [4] and CRH [11], but the mechanism was not known. CRH also triggered mast cell-dependent vasodilation in the microvasculature of human skin [35]. Interactions among CRH, NT, mast cells, and other cell types in the skin may represent the equivalent of the hypothalamic-pituitary-adrenal (HPA) axis outside the brain [36].

Here we show that NT induces VEGF release from human cultured mast cells, an action augmented by CRH, and blocked by a NTR antagonist. NT also induces CRHR-1, while CRH induces NTR gene expression in human mast cells. To the extent that CRH and NT are released under stress, the present findings represent a possible pathway that contributes to the worsening of inflammatory conditions with stress.

## Methods

### Peptides

CRH, NT and substance P (SP) were purchased from Sigma Aldrich (St. Louis, MO). The NTR antagonist, SR48692 was purchased from TOCRIS Bioscience (Ellisville, MO).

### Human Mast Cell Culture

LAD2 cells (kindly provided by Dr. A.S. Kirshenbaum, National Institutes of Health, NIH) were derived from a single patient with human mast cell leukemia. [37], [38] LAD2 cells were cultured in StemPro®-34 SFM medium (Invitrogen, Carlsbad, CA) supplemented with 100 U/mL penicillin/streptomycin and 100 ng/mL recombinant human stem cell factor (rhSCF) (Kindly supplied by Sweden Orphan Biovitrum AB, Stockholm, Sweden).

Human cord blood-derived cultured mast cells (hCBMCs) were cultured as described below. Human umbilical cord blood was collected following normal uncomplicated deliveries at Tufts Medical Center (Boston, MA) under Exemption #4 (discarded blood without identifiers) approved by the Tufts Medical Center Human Institutional Review Board (IRB). No consent form was required for discarded tissue. Cord blood cells were isolated by positive selection of CD34^+^/AC133^+^ cells with magnetic cell sorting using an AC133^+^ cell isolation kit (Milletnyi Biotec, Auburn, CA) as previously reported [39]. CD34^+^ cells were suspended in StemSpan serum-free expansion medium (StemCell Technologies, Vancouver, BC, Canada), supplemented with 100 ng/ml rhSCF; kindly supplied by Sweden Orphan Biovitrum AB, and 100 U/ml penicillin/100 µg/ml streptomycin (Invitrogen, Carlsbad, CA), and IL-3 (R&D Systems, Minneapolis, MN) for the first 3 weeks, and 50 ng/ml IL-6 (Peprotech, Rocky Hill, NJ) for 8 to 10 weeks. Fetal bovine serum (FBS; Invitrogen, Gibco, Carlsbad, CA) was added from week 6 on. The purity of hCBMCs was evaluated by immunocytochemical staining for tryptase [39]. hCBMCs cultured 7 to 10 weeks were used for the experiments.

### β-Hexosaminidase Assay

β-hexosaminidase (β-hex), as an index of mast cell degranulation, was assayed using a fluorometric assay. Briefly, hCBMCs and LAD2 cells (0.5×10^5^/tube) were stimulated, supernatant fluids were collected and cell pellets were lysed with 1% Triton X-100. Supernatant fluids and cell lysates were incubated in reaction buffer (p-nitrophenyl-N-acetyl-β-D-glucosaminide from Sigma) for 1 h and then 0.2 M glycine was added to stop the reaction. Absorbance was measured at 405 nm in an enzyme-linked immunosorbent assay reader, and the results are expressed as the percentage of β-hex released over the total.

### Cytosolic Calcium Measurements

Cytosolic calcium was measured in LAD2 cells at 37°C using Fura-2 (Invitrogen) as indicator. LAD2 cells were suspended in HEPES buffer (pH = 7.4) and were loaded with 30 nM Fura-2 AM for 20 min to allow Fura-2 to enter the cells. After centrifugation to remove excess dye, cells were resuspended in HEPES buffer at a concentration of 10^6^/ml and incubated for another 20 min. Cells were then transferred to 96-well plates (100 µl/well). NT was added at the indicated concentrations. Real-time Fura-2 fluorescence was read by MDC FlexStation II (Molecular Devices, Sunnyvale, CA) at an excitation wavelength of 340 nm/380 nm and emission wavelength of 510 nm. Results were analyzed according to the Invitrogen Fura-2 protocol and reported as the relative value of OD 340/380 nm as described previously [11].

### NTR Antagonism, Signaling Molecules and NFκB Assay

LAD2 mast cells were pretreated with/without the NTR antagonist (10 µM) SR 48692 (TOCRIS Bioscience, Ellisville, MO) for 30 min, then treated with NT (1 µM) for 5, 10, 20 min. Different signaling molecules were measured with PathScan® Sandwich ELISA Kit ( #7776, Cell Signaling Technology, Inc. Danvers, MA) and the absorbance was determined spectrophotometrically at 450 nm.

### Electromobility Gel Shift Assay (EMSA)

The nuclear extract of cultured mast cell was collected as previously described [40]. The consensus NF-κB oligonucleotide (AGTTGAGGGGACTTTCCCAGGC, Santa Cruz, CA) was radiolabeled by mixing 50 ng oligonucleotide, 70 µCi [γ-32P] ATP, 1 µl T4 polynucleotide kinase, 1.5 µl 10×T4 polynucleotide buffer and double-distilled water in a 15 µl reaction volume. The nuclear extract protein (10 µg) was then mixed with 0.5 ng [γ- 32P] ATP -labeled NF-kB oligonucleotide, 20 µg BSA, 2 µg pdI-dC, 2 µl Buffer D+ (20 mM Hepes pH 7.9, 20% glycerol, 100 mM KCl, 0.5 mM EDTA, 0.25% NP40), 4 µl Buffer F (20% FICOLL 400, 100 mM Hepes pH 7.9, 300 mM KCl) and DTT (2 mM) in 20 µl total volume at room temperature for 20 min. At the end of the reaction, the mixture was loaded on a non-denaturing 4% polyacrylamide gel using a running buffer containing 50 mM Tris pH 7.5, 0.38 M glycine and 2 mM EDTA, after which the gel was dried and exposed to Kodak x-ray film for autoradiography at −80°C.

### Quantitative PCR

Total RNA from cultured mast cells was isolated using RNeasy Mini Kit (Qiagen, Valencia, CA), according to the manufacturer's instructions. Reverse transcription was performed with 300 ng of total RNA using the iScript cDNA synthesis kit (BIO-RAD, Hercules, CA). In order to measure NT and NTR expression, quantitative real-time PCR was performed using Taqman gene expression assays (Applied Biosystems, Carlsbad, CA). The following probes obtained from Applied Biosystems were used: Hs00175048_m1 for NT, Hs00901551_m1 for NTSR1, Hs00366363_m1 for CRHR-1, and Hs00900055_m1 for VEGF. Samples were run at 45 cycles using Applied Biosystems 7300 Real-Time PCR System. Relative mRNA abundance was determined from standard curves run with each experiment. NT, NTR, CRHR-1, and VEGF gene expression was normalized to GAPDH (Hu, VIC TAMRA) endogenous control.

### VEGF Release Assay

Mast cells were treated with NT (1, 2 or 10 µM) for 24 h. VEGF release was measured by ELISA (R&D systems, Minneapolis, MN) in the supernatant fluid of control and stimulated LAD2 cells.

### Western Blot Analysis

Mast cells (40×10^6^ cells) were stimulated with NT or CRH (2 µM) for 24 h. The reaction was stopped by addition of ice-cold phosphate buffered saline (PBS). Cells were washed once with PBS and then lysed. Protein concentration of all fractions was determined by Bio-Rad protein assay with Polytron homogenizer (Kinematica Inc., Cincinnati, OH) in 500 µl of extraction buffer A (50 mM Tris, pH 7.4, 2 mM EGTA, 2 mM DTT, 0.5 mM PMSF, 1 µg/ml aprotinin, 1 µg/ml leupeptin). Equal amounts of protein were electrophoresed on 10% polyacrylamide gels and then transferred to a 0.44 µm Polyvinylidene fluoride membrane (Invitrogen). After blocking with 5% BSA, membranes were probed with antibody against CRHR-1 (Abcam, Cambridge, Mass Catalogue No. ab59023.) or NTR (Abcam, Catalogue No. ab117592) at 1∶1,000 dilution. Beta-actin was used as an internal control (Cell Signaling). For detection, the membranes were incubated with the appropriate secondary antibody conjugated to horseradish peroxidase (Cell Signaling) at 1∶1,000 dilution and the blots were visualized with enhanced chemiluminescence. Scanned densitometry and protein density calculation was performed by the National Institute of Health ImageJ.

### Fluorescence-Activated Cell Sorting (FACS)

Mast cells were treated with NT (1, 2, 10 µM) for 48 h, centrifuged at 500 g for 5 min and washed 3 times in PBS. Cells were blocked with 1 µg of mouse IgG1/human IgG per 10^5^ cells for 15 min at room temperature prior to staining. The Allophylocyanin (APC)-conjugated mouse monoclonal anti-human CRHR-1 antibody (R&D Systems, Minneapolis, MN) was added to stimulated cells that were then incubated for 45 min at 2–8°C. Following this incubation, unreacted anti-CRHR-1 conjugated antibody was removed by washing the cells 3 times with PBS. Finally, cells were resuspended in 400 µl of PBS for flow cytometric analysis. Cell surface expression of CRHR-1 was determined using 620–650 nm wavelength laser excitation and monitoring emitted fluorescence with a detector optimized to collect peak emissions at 660–670 nm (FACSCalibur flow cytometer, BD Biosciences, San Jose, CA).

### Statistical Analysis

All conditions were performed in triplicate, and all experiments were repeated at least three times (n = 3). Results are presented as mean±SD. Data from stimulated and control samples were compared using the unpaired 2-tailed, Student's *t*-test. Significance of comparisons is denoted by p<0.05.

## Results

### NT stimulates human mast cell degranulation

**Figure 1:**
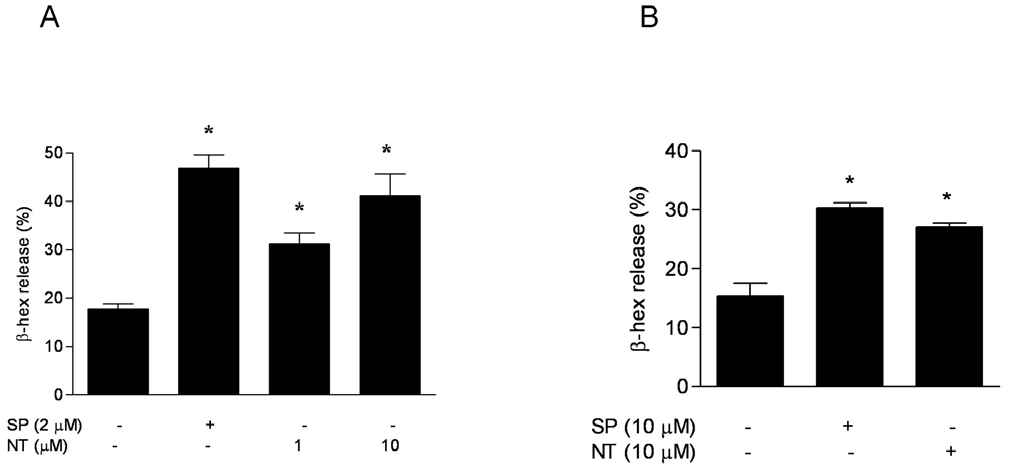
NT stimulates degranulation of human mast cells. (A) LAD2 mast cells and (B) hCBMCs were stimulated with NT for 30 min at 37°C. SP was used as a “positive” control to stimulate mast cell degranulation. The release of β-hexosaminidase (β-hex) was significantly elevated as compared to the control (n = 3; *p<0.01).

Here we show that NT (1 and 10 µM) triggered statistically significant degranulation (p<0.01) of human LAD2 mast cells as measured by β-hex secretion at 30 min (Fig. 1A). The effect of NT at 10 µM was comparable to that of the related peptide SP used at 2 µM as “positive” control (Fig. 1). To ensure that this finding was not limited to the use of the LAD2 leukemic mast cells, we repeated the same experiment using hCBMCs. hCBMCs secreted less β-hex and required a higher amount of both SP and NT (10 µM) to significantly (p<0.01) increase β-hex secretion as compared to the control (Fig. 1B).

### NT induces VEGF gene expression and release from LAD2 mast cells, augments the effect of CRH, and these actions are blocked by an NTR antagonist

**Figure 2:**
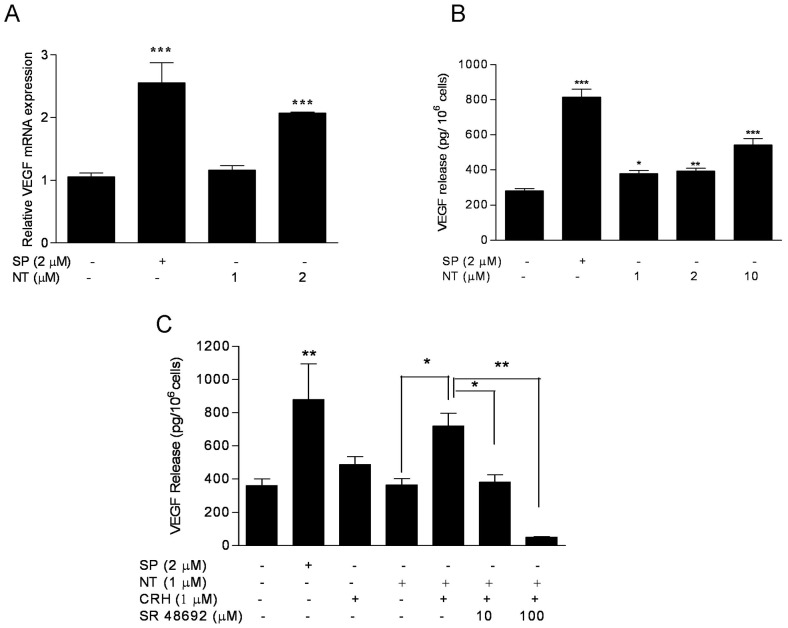
NT increases VEGF gene expression and protein secretion in human mast cells, which is blocked by a NTR antagonist. (A) VEGF mRNA expression was assessed following stimulation of LAD2 cells with NT (1, 2 µM) for 6 h; SP was used as a “positive” control to stimulate mast cell degranulation. (B) VEGF secretion from LAD2 cells was measured after stimulation with NT (1, 2, 10 µM) for 24 h; and (C) VEGF release from LAD2 cells treated for 48 hr with NT (1 µM) and/or followed with CRH (1 µM) for 24 h. The augmentation effect of NT and CRH is blocked by pre-treating with the NT antagonist SR 48692 (10 µM) for 30 min. For all experiments, n = 3; *p<0.05, **p<0.01, ***p<0.001 compared to control.

In view of the fact that NT can enhance the effect of CRH on skin vascular permeability [11], we investigated the effect of these peptides on VEGF release from LAD2 mast cells. NT (1, 2 µM) induced VEGF mRNA, with a significant 2-fold increase occurring at 6 h after treatment with 2 µM NT (Fig. 2A). The elevation of VEGF mRNA was sustained for 24 h (results not shown). To examine the effect of NT on VEGF protein secretion, LAD2 cells were treated with NT (1, 2, 10 µM) for 24 h. Stimulation with NT triggered a concentration-dependent release of VEGF, which was significant compared to control for NT concentrations ≥1 µM, with a maximum of 508 pg/10^6^ cells observed at 10 µM NT (Fig. 2B). Simultaneous addition of NT and CRH at concentrations (1 µM each) that had minimal effect on their own significantly increased the effect of CRH on VEGF release (Fig. 2C). In order to investigate if the effect of NT is specific, we pretreated LAD2 mast cells for 30 min with the specific NTR antagonist SR48692 (10 µM). This antagonist totally inhibited the combined effect of NT and CRH (Fig. 2C). Interestingly, SR48692 (10 µM) reduced even basal VEGF release (Fig. 2C).

### NT increases intracellular calcium and activates NF-κB in LAD2 mast cells

**Figure 3:**
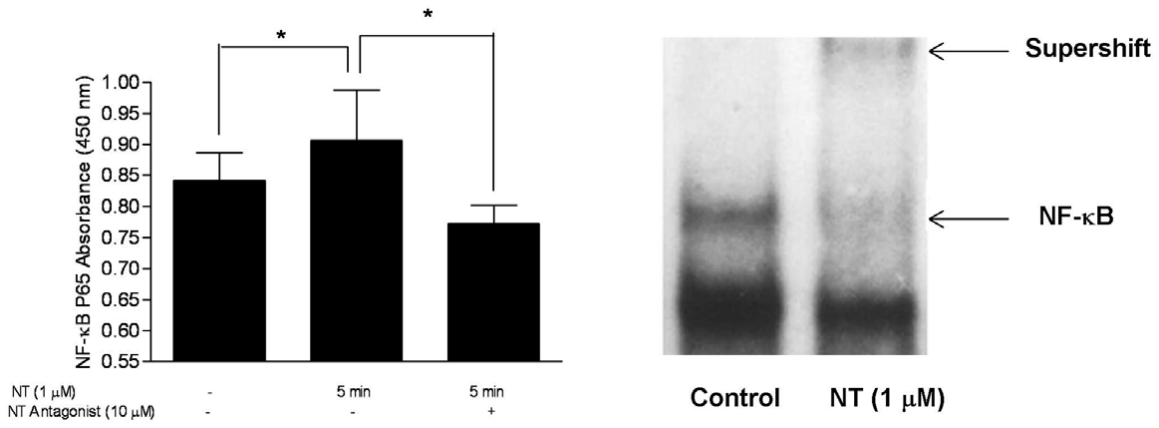
Effect of NT on NF-κB activation in human mast cells. LAD2 mast cells were pretreated with/without the NTR antagonist SR 48692 (TOCRIS Bioscience, Ellisville, MO) (10 µM) for 30 min, then treated with NT (1 µM) for 5, 10, 20 min. (A) Different molecules were measured with PathScan® Sandwich ELISA Kit (Cell Signaling Technology, Inc. Danvers, MA) and the absorbance for NF-κB was determined spectrophotometrically at 450 nm. (B) NF-κB was determined by EMSA.

We also investigated the involvement of a number of signaling molecules using the PathScan® Sandwich ELISA kit. There was no activation by NT of Phospho-SAPK/JNK (Thr183/Tyr185), p38 MARK, phospho-Stat3 (Tyr705) and phospho-IκB-α (Ser32). However, NT stimulated NFκB activation as evidenced by increased phospho NFκB p65 within 5 min of stimulation with NT; this activation was blocked by pretreatment with SR48692 (10 µM) for 30 min (Fig. 3A). NFκB activation was confirmed with EMSA (Fig. 3B).

Since an increase in intracellular calcium concentration is required for mast cell degranulation, we investigated the effect of NT on LAD2 cellular calcium levels. Continuous monitoring of stimulated cells with NT (1 µM) revealed a relative sustained increase of intracellular calcium levels compared to the unstimulated cells (Suppl. Fig. 1).

### NT induces CRHR-1 expression in human LAD2 mast cells as evidenced by Western blot and FACS analysis

**Figure 4:**
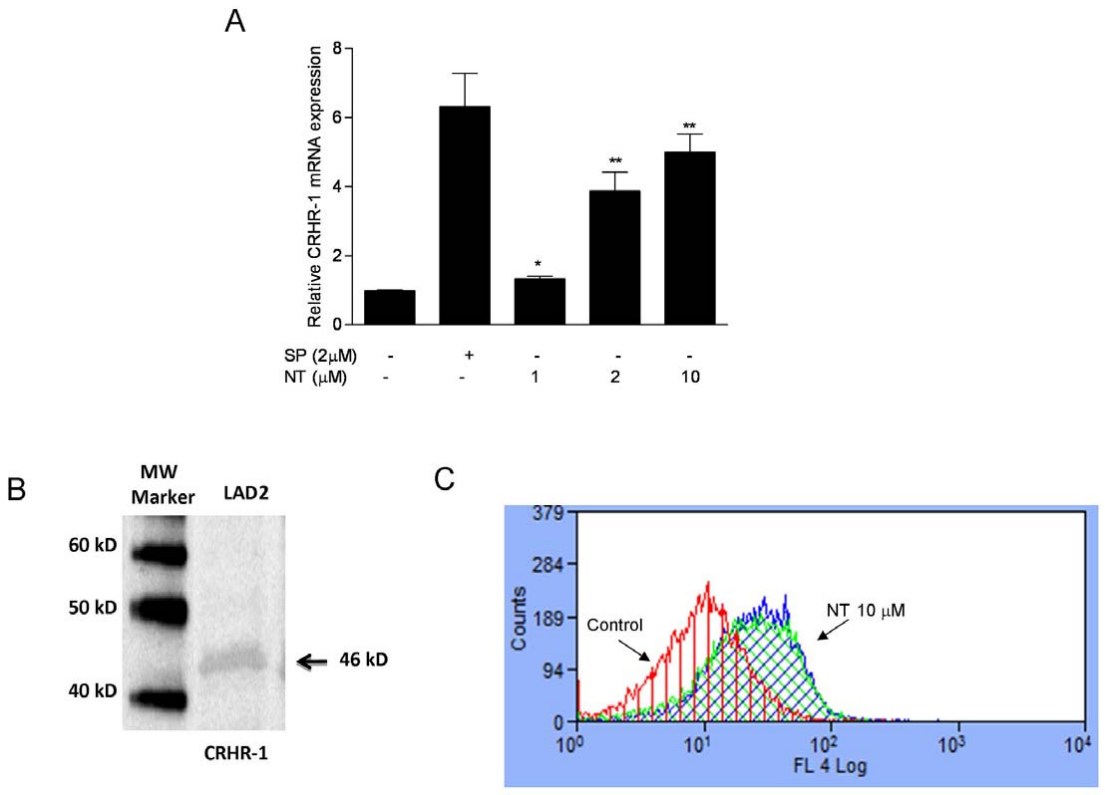
NT induces CRHR-1 expression in human mast cells. (A) LAD2 cells CRHR-1 gene expression was assessed following incubation with different concentrations of NT for 6 h. SP was used as a “positive” control to stimulate mast cell degranulation. Relative mRNA expression was measured by quantitative qPCR, normalized to GAPDH, and expressed relatively to the untreated cells (control). (B) Detection of CRHR-1 in LAD2 cells by Western blot analysis following incubation with NT (10 µM) for 24 or 48 h is shown. For all experiments, n = 3; *p<0.05, **p<0.01, compared to control and (C) CRHR-1 detection by FACS analysis in LAD2 cells following treatment with NT (1, 10 µM) for 48 h; y-axis indicates “counts” of cells, while the x-axis indicates log fluorescence intensity. For all experiments, n = 3; *p<0.05, **p<0.01, compared to control.

We next investigated how NT augments CRH-induced VEGF release. We felt that one possibility might be the ability of NT to induce CRHR-1 expression in LAD2 mast cells. Stimulation with NT (1, 2, 10 µM) for 6 h significantly increased relative CRHR-1 mRNA expression compared to control cells, with a 4-fold increase noted for 2 µM and 5-fold increase for 10 µM (Fig. 4A). Stimulation of hCBMCs with NT (10 µM) also induced a more than 2.5-fold increase in relative CRHR-1 mRNA expression (Suppl. Fig. 2). Expression of CRHR-1 protein in LAD2 cells stimulated with NT (1 µM) was confirmed by Western blot-analysis (Fig. 4B), showing a predominant band of about 46 kDa, (a single band of 47 kDa was also detected in HaCaT cells as reported previously [41]). The specificity of the response was confirmed by pre-incubation of the CRHR-1 Ab with a CRHR-1 blocking peptide, following which the band disappeared, confirming that the band was specific for CRHR-1 (results not shown). The presence of CRHR-1 on the cell surface at 48 h was confirmed by FACS analysis (Fig. 4C).

### CRH induces NT and NTR gene expression in human mast cells

**Figure 5:**
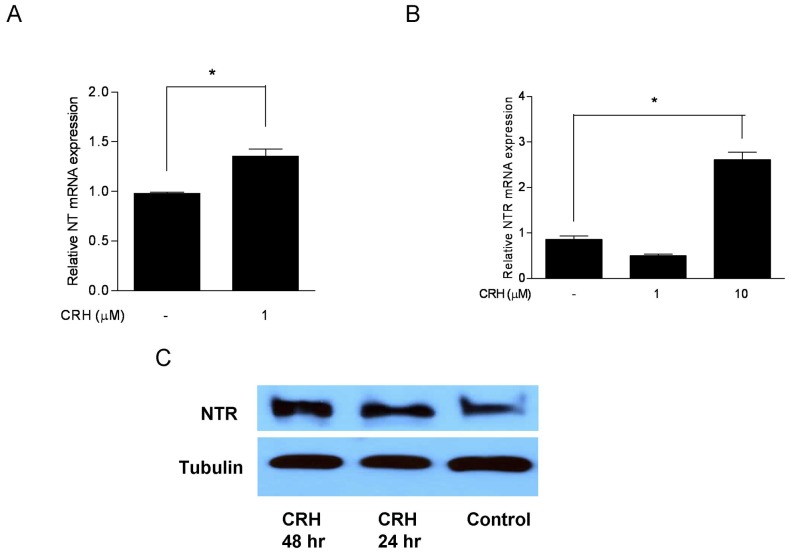
CRH induces NT and NTR gene expression in human mast cells. (A) NT and (B) NTR gene expression in LAD2 cells following incubation with the indicated concentrations of CRH for 6 h. Relative mRNA expression was measured by quantitative qPCR, normalized to GAPDH, and expressed relatively to the untreated cells (control). (C) Western blot analysis of NTR following incubation with CRH (10 µM) for 24 h. Tubulin was used as an internal control. For all the experiments, n = 3; *p<0.05, **p<0.01, compared to control.

We also investigated the effect of CRH on NT and NTR gene expression on LAD2 mast cells. Addition of CRH (1 µM) to LAD2 cells significantly induced NT gene expression (Fig. 5A). CRH (1, 10 µM) also induced NTR gene expression (Fig. 5B). The presence of NTR protein was confirmed with Western blot analysis (Fig. 5C). Similar results were obtained using hCBMCs (Suppl. Fig. 3). Pretreatment of LAD2 cells with the CRHR-1 antagonist Antalarmin (10 µM) for 60 min prion to CRH (1 µM) inhibited NT and NTR gene expression (results not shown).

## Discussion

This is the first report to our knowledge showing that NT stimulates human mast cells to release VEGF and augments the effect of CRH on VEGF release through a NTR specific manner. The ability of the NTR antagonist, SR48692, to block the effect of NT is consistent with the fact that mast cells express NTR [8]. Interestingly, the NTR antagonist reduced even basal VEGF release suggesting that mast cell-derived NT has a possible autocrine effect. This finding is supported by a recent report that the human leukemic mast cells (HMC-1) can synthesize NT precursor and release NT-like peptides [42]. Our present findings offer one possible explanation for our previous report that NT augments the ability of CRH to increase skin vascular permeability in rodents [11].

The concentrations of NT used in this study were fairly high, but not unlike what had previously been used for histamine release from rodent peritoneal mast cells [7], [43], as well as to increase vascular permeability in rat skin [5] and in skin blisters in a mast cell-dependent manner [4], [6]. The high NT concentration necessary may be due to either the lack of sufficient number of NTR on the human mast cells used or the fact that NT is rapidly degraded during incubation as shown by stimulated rat mast cells [10]. In fact, mast cells were considered as negative regulators of NT-induced septic shock in mice [44].

We also show, by qPCR, Western blot and FACS analysis, that NT induces CRHR-1 gene expression. We recently reported that the related peptide SP also induced CRHR-1 expression in human mast cells [45]. The effect of NT on CRHR-1 gene expression may be partly due to histamine release since histamine induced the expression of CRHR-1α in normal human endothelial and synovial cells [46]. Interestingly, CRH induces NT and NTR gene expression in human mast cells implying that there is reciprocal activation of the two stress-related neuropeptides.

The mechanism through which NT stimulates mast cell degranulation and VEGF release, as well as CRHR-1 expression is not yet clear. It is at least NTR specific, as it is blocked by a NTR antagonist, and requires calcium. There is also some evidence that cationic peptides such as NT may bypass mast cell surface receptors and act on a G protein directly [47], [48]. For instance, SP [49] was shown to act directly on G proteins, “mimicking” the action of ligand binding to specific receptors. NT and SP may interact with each other's receptor [50] since, SP-induced skin vascular leakage was blocked by N-acetyl-NT [51] and NT-induced colonic mast cell degranulation was blocked by a SP receptor antagonist [52].

Even though NT stimulated NF-κB activation as evidenced by increased phospho NF-κB p65 and electromobility gel shift assay, it is not definitive that NF-κB activation is mandatory. Knockdown of NF-κB p65 with siRNA will be required to conclusively prove NF-κB requirement. NT had previously been shown to increase expression of EGF receptor [53] through NF-κB [54]. These results imply that the effect of NT may vary depending on the number of NTR or CRHR expressed and/or the tissue type.

However, the action of NT does not seem to involve phospho-SAPK/JNK, p38 MARK, phospho-Stat3 or phosph-IκB-α. A possible explanation of how NT induces CRHR-1 may be nuclear translocation of some element that activates transcription of the respective receptor gene. For instance, it was recently shown that dexamethasone increased synthesis and nuclear translocation of ReIB/NF-κB-2, an endogenous stimulatory signal that increases CRH synthesis [55].

CRH is typically secreted from the hypothalamus under stress and activates the HPA axis [56]. CRH and CRHR gene and peptide expression has been documented in human skin [57], [58]. These findings have led to the proposal that the skin may have its own equivalent of the HPA axis [36], [59]. Stress induces local release of CRH in the skin [60], and stimulates degranulation of skin mast cells [4] a portion of which expresses CRHR-1 [61]. Mast cells are located close to CRH-positive nerve endings [62], and are juxtaposed to nerve endings during hair follicle formation [63]. In fact, CRH stimulates the development of mast cells from hair follicle precursors [64]. CRH can be released from nerve endings [57], but it is also synthesized by skin cells [65], immune cells [66], and mast cells [67]. Human mast cells also express mRNA and protein for CRHR-1, activation of which induces selective release of VEGF [68]. An isoform of VEGF actually is vasodilatory [69], [70] and can contribute to increased skin vascular permeability. In fact, skin mast cells have been considered as a potential stimulus for neovascularization [71].

Stress worsens allergic diseases in general [17]–[20], as well as skin diseases such as AD [21]–[24] a chronic skin inflammatory disease [26], [28] strongly associated with other atopic disorders [13] such as asthma and allergic rhinitis [29]. Mast cells and nerve-mast cell contacts are increased in lesional AD skin [30]–[32]. Our results provide evidence for a possible mechanism through which stress increases immune responses. In addition, CRH decreases IL-10 production from Treg cells in AD patients [72]. IL-10 also suppresses mast cell IgE receptor expression and IgE-mediated anaphylaxis [73], two possible mechanisms through which stress exacerbates immune responses. We recently showed that CRH leads to down-regulation of CRHR-1 gene expression in lesional skin of AD patents [74], suggesting chronic overstimulation.

Mast cells are critical in allergy and innate immunity in the skin [13], [75], as well as in AD [71], [76]–[78]. They may, in fact, both promote and regulate skin inflammation [79]. The present findings suggest that NT can induce CRHR-1 expression and CRH can induce NTR expression on mast cells (Suppl. Fig. 4). Activation of CRHR-1 and NTR on mast cells will result in release of VEGF. However, NTR activation also leads to release of IL-8 and TNF, which can promote inflammation. Release of neurosensitizing molecules (e.g. ATP, histamine, kinins, PGD_2_, and tryptase) can also induce inflammation, while release of VEGF can both promote inflammation and wound healing depending on the timing and the receptors expressed. In other words, NT may induce inflammation through its immediate action, but may then promote healing through its induction of CRHR-1 expression and VEGF release (Suppl. Fig. 4). Interactions among NT, CRH, and mast cells may mediate the effect of stress on immunity by at least targeting mast cells [80], as part of a “brain-skin” connection [16]. The possible role of CRH in immune-mediated skin diseases was reviewed recently [81], [82].

NTR and/or CRHR-1 antagonists, as well as mast cell blockers, could provide possible therapeutic approaches for stress-induced inflammatory diseases.

## Supporting Information

Figure S1 **Effect of NT on intracellular calcium levels in human mast cells.** Intracellular calcium levels were measured in LAD2 cells using Fura-2 and are expressed as the 340/380 nm absorbance ratio. Intracellular calcium levels were monitored continuously for 20 min at 37°C after stimulation with NT (1, 10 µM).
(TIF)

Figure S2 **NT induces CRHR-1 expression in hCBMCs.** hCBMCs CRHR-1 gene expression was assessed following incubation with different concentrations of NT for 6 h. SP was used as a “positive” control to stimulate mast cell degranulation. Relative mRNA expression was measured by quantitative qPCR, normalized to GAPDH, and expressed relatively to the untreated cells (control).
(TIF)

Figure S3 **CRH induces NT and NTR gene expression in hCBMC.** hCBMCs (A) NT and (B) NTR gene expression following incubation with the indicated concentrations of CRH for 6 h. Relative mRNA expression was measured by quantitative qPCR, normalized to GAPDH, and expressed relatively to the untreated cells (control).
(TIF)

Figure S4 **Diagrammatic representation of the proposed steps based on our results.** Circulating CRH and NT, possibly released from dorsal root ganglia, induce expression of their respective receptors on mast cells. Stimulation of these receptors leads to synergistic mast cell activation and release of TNF and VEGF, which facilitate inflammation and keratinocyte proliferation, as well as neurosensitizing molecules that contribute to pruritus.
(TIF)
