# Supplementary figures and images for: Neurotensin and CRH Interactions Augment Human Mast Cell Activation
Source: PLoS One. 2012 Nov 14;7(11):e48934. doi: 10.1371/journal.pone.0048934 (PMC3498358; doi:10.1371/journal.pone.0048934)

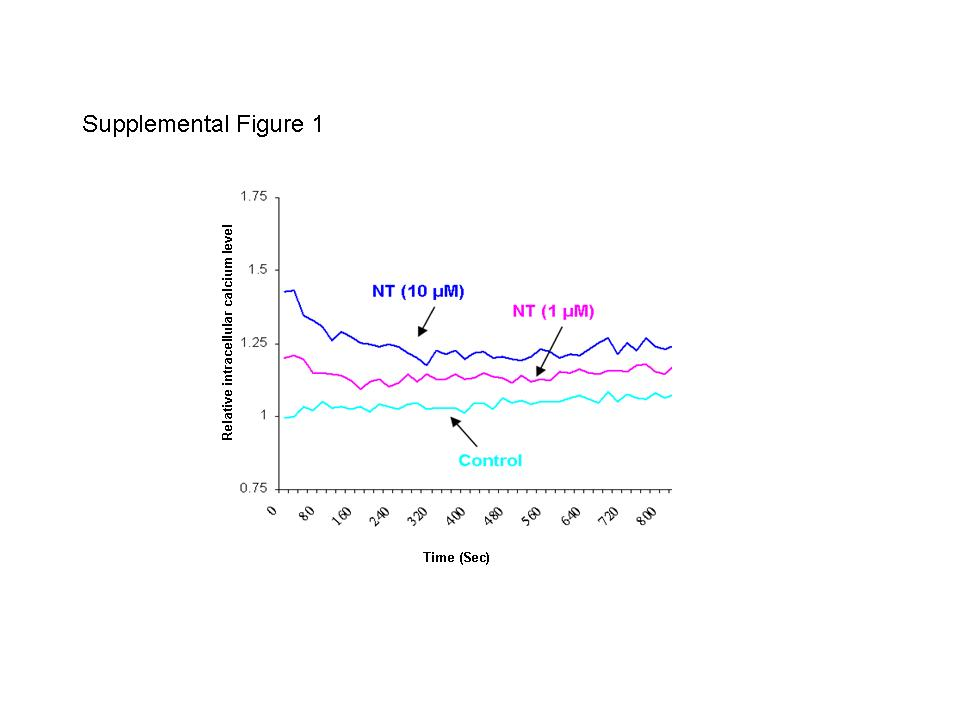

Supplement: Figure S1 — Effect of NT on intracellular calcium levels in human mast cells. Intracellular calcium levels were measured in LAD2 cells using Fura-2 and are expressed as the 340/380 nm absorbance ratio. Intracellular calcium levels were monitored continuously for 20 min at 37°C after stimulation with NT (1, 10 µM). (TIF) [file pone.0048934.s001.tif]

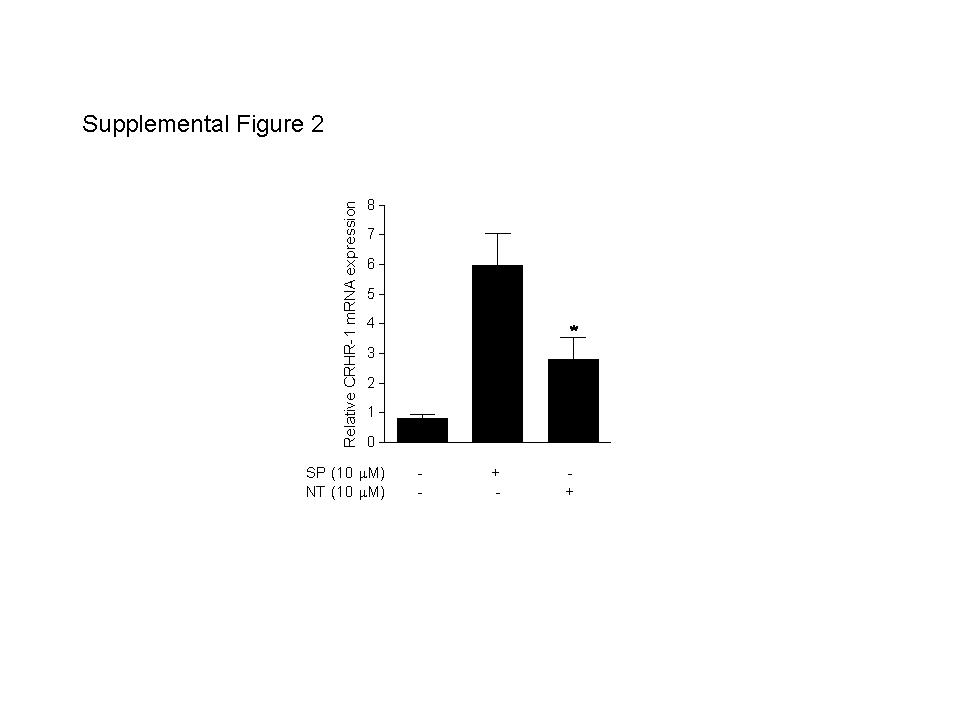

Supplement: Figure S2 — NT induces CRHR-1 expression in hCBMCs. hCBMCs CRHR-1 gene expression was assessed following incubation with different concentrations of NT for 6 h. SP was used as a “positive” control to stimulate mast cell degranulation. Relative mRNA expression was measured by quantitative qPCR, normalized to GAPDH, and expressed relatively to the untreated cells (control). (TIF) [file pone.0048934.s002.tif]

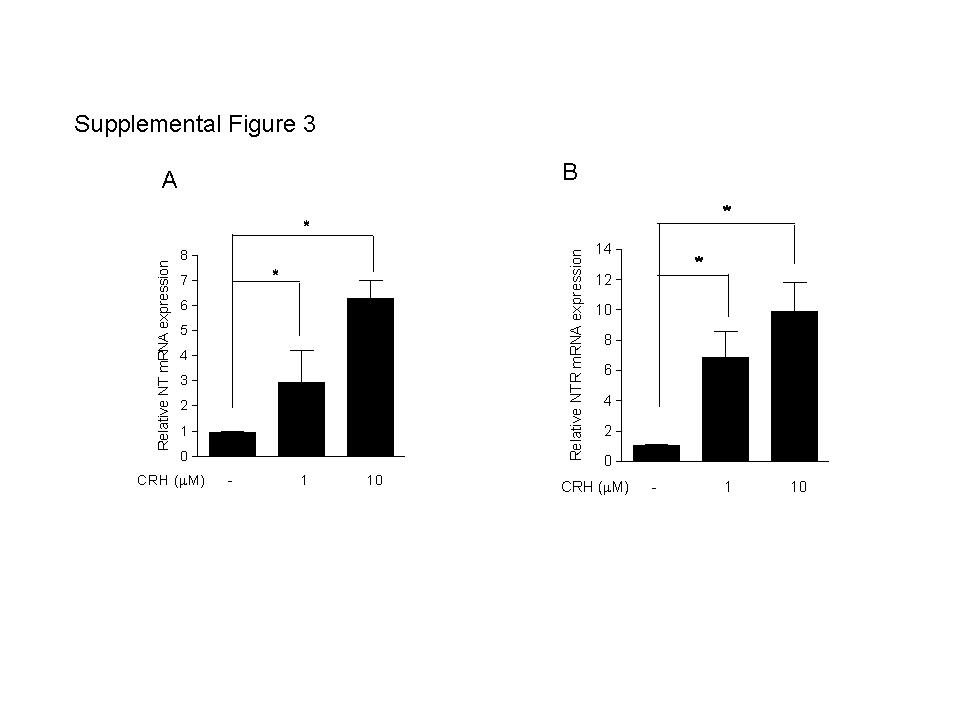

Supplement: Figure S3 — CRH induces NT and NTR gene expression in hCBMC. hCBMCs (A) NT and (B) NTR gene expression following incubation with the indicated concentrations of CRH for 6 h. Relative mRNA expression was measured by quantitative qPCR, normalized to GAPDH, and expressed relatively to the untreated cells (control). (TIF) [file pone.0048934.s003.tif]

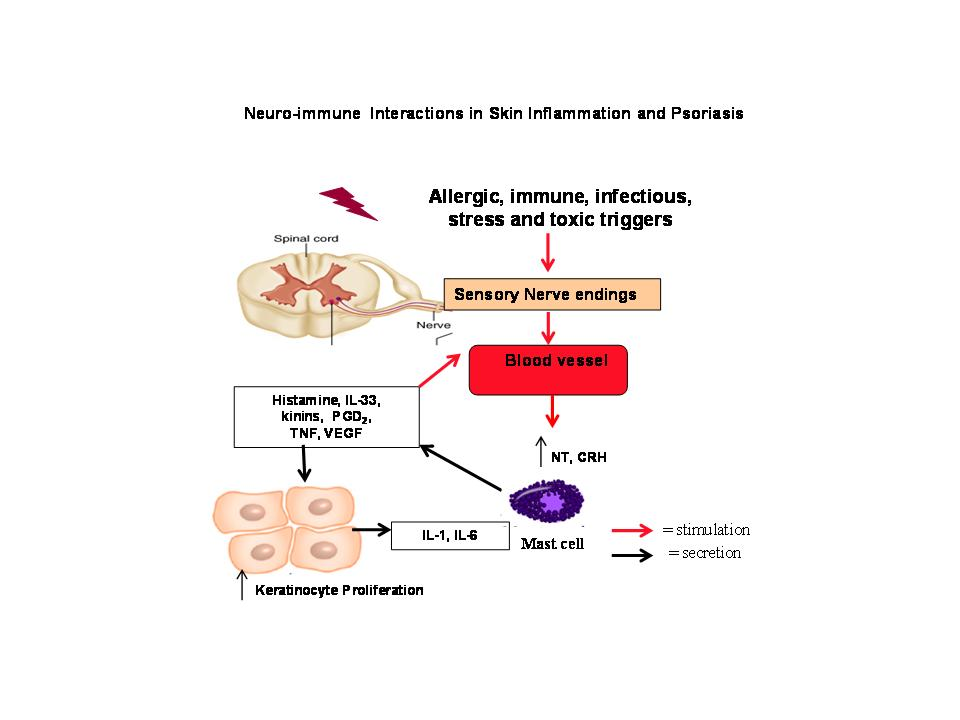

Supplement: Figure S4 — Diagrammatic representation of the proposed steps based on our results. Circulating CRH and NT, possibly released from dorsal root ganglia, induce expression of their respective receptors on mast cells. Stimulation of these receptors leads to synergistic mast cell activation and release of TNF and VEGF, which facilitate inflammation and keratinocyte proliferation, as well as neurosensitizing molecules that contribute to pruritus. (TIF) [file pone.0048934.s004.tif]
